# Development of marker-free transgenic *Jatropha *plants with increased levels of seed oleic acid

**DOI:** 10.1186/1754-6834-5-10

**Published:** 2012-02-29

**Authors:** Jing Qu, Hui-Zhu Mao, Wen Chen, Shi-Qiang Gao, Ya-Nan Bai, Yan-Wei Sun, Yun-Feng Geng, Jian Ye

**Affiliations:** 1 Temasek Life Sciences Laboratory, 1 Research Link, National University of Singapore, Singapore

**Keywords:** Biodiesel, Cre-lox recombination, FAD2, high oleic acid, *Jatropha*, marker free, transgenic

## Abstract

**Background:**

*Jatropha curcas *is recognized as a new energy crop due to the presence of the high amount of oil in its seeds that can be converted into biodiesel. The quality and performance of the biodiesel depends on the chemical composition of the fatty acids present in the oil. The fatty acids profile of the oil has a direct impact on ignition quality, heat of combustion and oxidative stability. An ideal biodiesel composition should have more monounsaturated fatty acids and less polyunsaturated acids. Jatropha seed oil contains 30% to 50% polyunsaturated fatty acids (mainly linoleic acid) which negatively impacts the oxidative stability and causes high rate of nitrogen oxides emission.

**Results:**

The enzyme 1-acyl-2-oleoyl-sn-glycero-3-phosphocholine delta 12-desaturase (FAD2) is the key enzyme responsible for the production of linoleic acid in plants. We identified three putative *delta 12 fatty acid desaturase *genes in *Jatropha *(*JcFAD2s*) through genome-wide analysis and downregulated the expression of one of these genes, *JcFAD2-1*, in a seed-specific manner by RNA interference technology. The resulting *JcFAD2-1 *RNA interference transgenic plants showed a dramatic increase of oleic acid (> 78%) and a corresponding reduction in polyunsaturated fatty acids (< 3%) in its seed oil. The control *Jatropha *had around 37% oleic acid and 41% polyunsaturated fatty acids. This indicates that FAD2-1 is the major enzyme responsible for converting oleic acid to linoleic acid in *Jatropha*. Due to the changes in the fatty acids profile, the oil of the *JcFAD2-1 *RNA interference seed was estimated to yield a cetane number as high as 60.2, which is similar to the required cetane number for conventional premium diesel fuels (60) in Europe. The presence of high seed oleic acid did not have a negative impact on other *Jatropha *agronomic traits based on our preliminary data of the original plants under greenhouse conditions. Further, we developed a marker-free system to generate the transgenic *Jatropha *that will help reduce public concerns for environmental issues surrounding genetically modified plants.

**Conclusion:**

In this study we produced seed-specific *JcFAD2-1 *RNA interference transgenic *Jatropha *without a selectable marker. We successfully increased the proportion of oleic acid versus linoleic in *Jatropha *through genetic engineering, enhancing the quality of its oil.

## Background

The depletion of world petroleum reserves, increasing prices and the rising pollution concerns has stimulated the search for alternative renewable fuel sources. Biodiesel from plants is rapidly emerging as the alternative to petroleum fuel. The fuel properties of biodiesel are highly dependent on the composition of the fatty acid mixture in the oil. The majority of plant oils are generally composed of five common fatty acids: palmitate (16:0), stearate (18:0), oleate (18:1), linoleate (18:2) and linolenate (18:3) [1]. Palmitate and stearate are saturated fatty acids, oleate a monounsaturated fatty acid and linoleate and linolenate are polyunsaturated fatty acids.

It is known that biodiesel with high monounsaturated fatty acid content (oleate) has excellent characteristics with respect to ignition quality, nitrogen oxides (NO_x_) emissions and fuel stability [2]. However, most plant oils used as biodiesel feedstock have a high level of polyunsaturated fatty acids (linoleate and linolenate acids) which negatively impacts the biodiesel quality. This is because the fatty acid desaturation decreases the biodiesel stability affecting the cetane number (CN). The CN value is perhaps the most important factor for biodiesel since it is a determinant parameter for the ignition quality of diesel fuels and also negatively correlates with NO_x _emissions. Esters of linoleate are 49 times more reactive than oleate esters because the former contain an easily oxidized bis-allylic methylene group between the two double bonds [3]. The oxidation of polyunsaturated esters of linoleate results in the initial accumulation of hydroperoxides, which eventually polymerize to form insoluble sediments that are capable of plugging filters, fouling injectors and interfering with engine performance and safety. Moreover, the CN of methyl oleate is higher than the minimal biodiesel standard [1]. Hence the biodiesel derived from plant oil with high oleate content is expected to have better fuel qualities. This has been shown in soybean, where lines with high levels of oleic acid and low levels of polyunsaturated fatty acids were developed using a transgenic strategy that downregulates the expression of *fatty acid desaturase 2 *(*FAD2*) gene [2,4]. As predicted, biodiesel derived from these high oleic soybean oils displayed improved fuel characteristics with regard to fuel stability and NO_x _missions [2,4].

The demand for biodiesel soared in recent years because of government subsidies and mandates. However, the increased plantation of food crops, such as corn and soy for biofuel, exerts pressure on food production in terms of arable land use and drives up the prices of vegetable oil. One way to ease this concern is to make use of new or under-utilized non-edible oilseed crop that can grow on marginal land [5] not suitable for food crops. The characteristics of *Jatropha curcas *make it a suitable candidate for a biofuel crop. It is a non-food crop with high oil content and it can grow on degraded soils and waste lands unsuitable for food crops.

*Jatropha *seeds contain 30% to 50% polyunsaturated fatty acids (mainly linoleic acid) [6]. To develop *Jatropha *as a premium sustainable bioenergy crop there is a need to improve its fatty acid profile. Theoretically it is possible to produce plants with high level of oleic acid through conventional breeding by exploiting the variation found in the *Jatropha *germplasm. But the high oleic acid germplasms are always associated with a lesion in one of the *FAD2 *genes, which plays an important role in environmental adaptation during vegetative growth [7]. Hence finding a suitable germplasm may not be easy. Alternatively, transgenic technology can be used to increase the oleic acid content of *Jatropha *oil. Previously, we have identified the function of several key *Jatropha *genes that regulate fatty acid chain length and saturation levels [8]. Among these, the *JcFAD2-1 *gene is most important as it mediates the conversion of oleic acid to linoleic acid. By suppressing its expression in a seed-specific manner using RNA interference (RNAi) we were able to generate lines showing increased levels of oleic acid and an estimated CN as high as 60.2. Although several genetic transformation methods have been reported for *Jatropha *[9,10], this is the first report of using transgenic technology to improve its agronomic traits and seed oil quality. To increase the public acceptance of such transgenic plants we developed a transformation procedure that uses the chemical inducible Cre-lox system to obtain marker-free transgenic *Jatropha*.

## Results

### Identification of *FAD2 *genes

The first step towards generating high oleic acid *Jatropha *was to determine the gene(s) which encode putative microsomal delta 12 fatty acid desaturase. For this, we isolated three cDNAs which possess extensive similarity to extant *FAD2 *genes from a *J. curcas *seed cDNA library (ZC Yin *et al*., unpublished data). One of the cDNA had a mutation that generated a premature stop codon, rendering it non-functional. The other two cDNAs encoded proteins of 383 and 387 amino acids that were 74% identical to each other and 77.3% and 72.1% identical to *Arabidopsis *FAD2, respectively (Additional file 1). We further confirmed that there are at least three *FAD2 *genes in the *Jatropha *genome through the data mining of both deep sequencing data of Temasek Life Sciences Laboratory (H Yan, unpublished data) and the *Jatropha *genome database available online [11]. The cDNA which encoded a higher amino acid sequence identity to the FAD2 enzyme family was designated as *JcFAD2-1 *[GenBank: JN544421] and another one was named *JcFAD2-2 *[GenBank: JN544422]. The non-functional one shared higher similarity with *JcFAD2-2 *and was named as *JcFAD2-2m*. JcFAD2-1 has identical amino acid sequences to *Arabidopsis thaliana *FAD2 at its enzyme active center in three conserved His-rich boxes (data not shown), while JcFAD2-2 has a variation on a key residue Ala in active site His-rich box 3 (Thr in JcFAD2-2; Additional file 2). The change of small hydrophobic Ala substituted with polar Thr could potentially alter FAD2-2 substrate specificity and enzyme activity because the hydrophobic core environment is crucial for its activity [12].

To investigate the gene expression patterns of *FAD2-1 *and *FAD2-2*, RNA was extracted from different stages of seed development (three weeks, five weeks, seven weeks and eight weeks after fertilization, corresponding to the early, middle, late and mature stages of *Jatropha *seed) and used in RT-PCR reactions containing primers specific for each cDNA. As shown in Additional file 2, the expression of *FAD2-1 *gene is high in seeds and weaker in vegetative tissues, while the *FAD2-2 *gene is expressed highly in seeds and not detectable in leaf tissue. The expression pattern of these two *FAD2 *genes in *Jatropha *is very similar to others in the Euphorbiaceae: *FAD2 *and Fatty acid hydroxylase 12 (*FAH12*) in castor bean [13], *FAD2 *and *FADX *in the tung tree (*Aleurites fordii*) [12]. All the above data suggest that JcFAD2-2, similar to FADX and FAH12, may function more like an unusual fatty acid enzyme than a desaturase. We have previously identified that JcFAD2-1 mediates the conversion of oleic acid to linoleic acid in *Jatropha *leaf by virus-induced gene silencing (VIGS). Therefore, in this study we chose *FAD2-1 *as our target to knockdown for the development of high oleic acid composition.

### Generation of marker-free FAD2-1i RNAi transgenic *Jatropha *using a constitutive promoter and analysis of oleic content in T0 leaves

**Figure 1:**
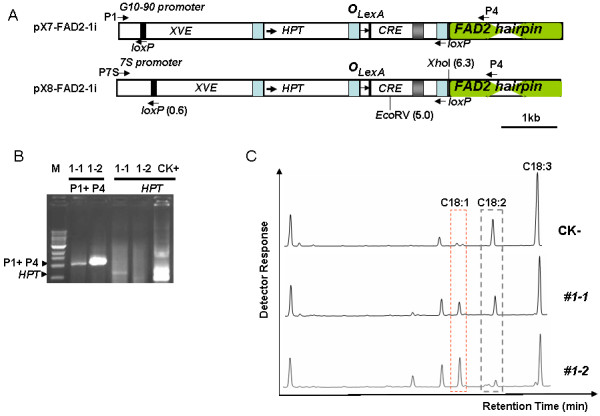
**Verification of the inducible marker-free and RNAi system in *Jatropha***. **(A) S**chematic diagram showing structure features of the inducible pX7-FAD2-1i and pX8-FAD2-1i constructs and Cre/loxP-mediated DNA recombination. For details, see [14,15]. **(B) **Genotypic analysis of T0 transgenic shoots #1-1 and #1-2. The plasmid DNA of pX7-FAD2-1i containing *HPT *gene was used as positive control (CK+). **(C) **GC analysis of fatty acid composition in T0 transgenic shoots #1-1, #1-2 and untransformed shoot (CK-).

Considering the possible large-scale commercial planting of such transgenics as *Jatropha*, we aimed to obtain transgenic plants free of an antibiotic selection marker. To accomplish this, we used a chemical inducible Cre-lox-mediated site-specific recombination system which had been shown to work in *Arabidopsis *[14,15]. To produce transgenic *Jatropha *plants with high oleic acid content we used RNAi technology to silence *JcFAD2-1 *expression by targeting an 862 bp coding region that shares 72.9% identity with *JcFAD2-2*. We first obtained hygromycin-resistant regenerated shoots. Once the small shoots (2 to 3 mm) came out, they were immediately transferred to β-estradiol induction medium without hygromycin to induce marker excision. After two weeks of induction, shoots were transferred to regeneration medium II without hygromycin. Figure 1A shows the chemical inducible RNAi construct used to silence the *JcFAD2-1 *gene. Upon induction, Cre-lox mediated recombination excises the hygromycin gene-containing DNA fragment. Only this recombination event happened; the *JcFAD2-1 *hairpin structure is situated immediately downstream of the G10-90 promoter (pX7-FAD2-1i). The G10-90 promoter drives constitutive expression and has higher expression levels than does the cauliflower mosaic virus 35S promoter in *Arabidopsis*. It drives the constitutive expression of *JcFAD2-1 *hairpin RNA, which is further processed into *JcFAD2-1 *specific siRNA. Finally, these siRNA guide the degradation of *JcFAD2-1 *transcripts.

We randomly selected ten regenerated shoots of pX7-FAD2-1i and extracted their genomic DNAs for genotypic analysis. PCR analysis revealed a smaller fragment of expected size in two out of ten regenerated shoots consistent with successful marker excision (Figure 1B). One of the lines, #1-1, had a band corresponding to the *hygromycin phosphotransferase *gene (*HPT*) suggesting that the shoot was chimeric marker free. By contrast, no band corresponding to the *HPT *gene was detected in the other line, #1-2, suggesting that line #1-2 might be a pure marker-free transgenic *Jatropha *(Figure 1B).

In *Arabidopsis, FAD2 *encodes the desaturase responsible for the introduction of a second double (Δ^12^) bond in oleate. To confirm that RNAi-mediated suppression of *FAD2-1 *should block the conversion of 18:1 to 18:2 fatty acids in *Jatropha*, we used gas chromatography (GC) of fatty acid methyl ester (FAME) to analyze leaf fatty acid profiles. As predicted, there was a higher oleic acid content in line #1-1 and a much higher level of oleic acid in line #1-2 compared with regenerated shoots from control (untransformed) cotyledons (Figure 1C). The linoleic acid level was significantly reduced in lines #1-1 and #1-2, suggesting that suppression of *FAD2-1 *increased the oleic acid level at the expense of linoleic acid and that the *JcFAD2-1 *encoded enzyme mediates conversion of oleic acid to linoleic acid.

### Characterization of oleic acid content in seeds and leaves of T1 pX7-FAD2-1 transgenic RNAi lines

**Figure 2:**
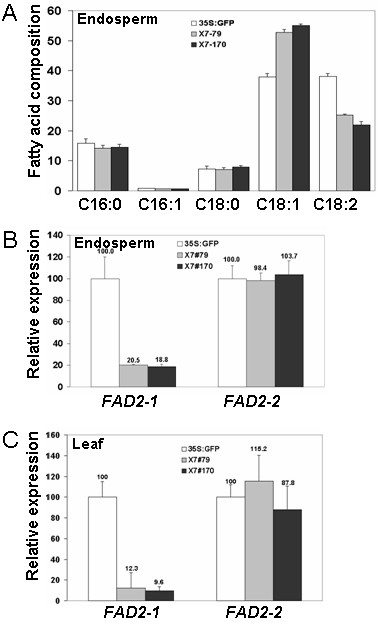
**Molecular and oil composition analysis of X7-FAD2-1i lines**. **(A) **GC analysis of fatty acid composition in T1 endosperms of #79, #170 and 35S:GFP (CK-) lines. **(B) **Quantitative analysis of *FAD2-1 *and *FAD2-2 *expression levels in T1 endosperm of #79, #170 and 35S:GFP (CK-) lines. **(C) **Quantitative analysis of *FAD2-1 *and *FAD2-2 *expression levels in T1 leaves of #79, #170 and 35S:GFP (CK-) lines.

Using PCR analysis, we identified 20 more putative pure marker-free X7-FAD2-1 RNAi lines (partial genotyping results shown in Additional file 3). These transgenic lines were grown to maturity in a greenhouse. T1 seeds were collected and endosperms were carefully separated from the embryos germinated on hormone-free medium. GC analysis showed that oleic acid content in the best two lines, X7#79 and X7#170, increased to 50% to 60% of the total fatty acid in contrast to the 36.7% in control (35S:GFP) endosperms (Figure 2A). Correspondingly, the linoleic acid levels reduced to less than 25% from the original 41% in control endosperms (Figure 2A). We noted that the increase of the oleic acid level was moderate and not as high as that found in the initial T0 leaves (#1-2 in Figure 1C) or in *Jatropha *leaves treated with Tobacco Rattle Virus-induced *FAD2-1 *RNAi during our previous VIGS studies [8]. One possibility could be the weak expression of the G10-90 promoter in *Jatropha *seeds. This assumption was confirmed by RNA analysis using quantitative RT-PCR, which showed that the two transgenic lines still expressed *FAD2-1 *transcripts at 20% of the control level (Figure 2B). It also showed that the *FAD2-1 *suppression was gene-specific as there was no effect on the expression of *FAD2-2 *in the endosperms of these two transgenic lines (Figure 2B). We also analyzed the *FAD2-1 *RNAi effect in vegetative organs of T1 plants. There was considerable reduction of *FAD2-1 *expression levels in T1 leaves as well (Figure 2C).

### High oleic acid transgenic lines with seed-specific promoter

**Figure 3:**
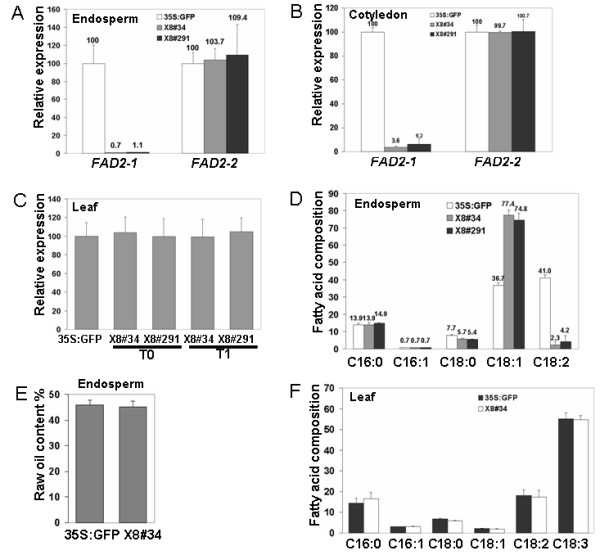
**Molecular and oil composition analysis of X8-FAD2-1i lines**. **(A) **Quantitative analysis of *FAD2-1 *and *FAD2-2 *expression levels in T1 endosperm of #34, #291 and 35S:GFP (CK-) lines. **(B) **Quantitative analysis of *FAD2-1 *and *FAD2-2 *expression levels in T1 cotyledons of #34, #291 and 35S:GFP (CK-) lines. **(C) **Quantitative analysis of *FAD2-1 *expression levels in T0 and T1 true leaves of #34, #291 and 35S:GFP (CK-) lines. **(D) **GC analysis of fatty acid composition in T1 endosperms of #34, #291 and 35S:GFP (CK-) lines. **(E) **Oil content analysis in T1 endosperm of #34 and 35S:GFP (CK-) lines. **(F) **GC analysis of fatty acid composition in T1 true leaves of #34 and 35S:GFP (CK-) lines.

Since the G10-90 promoter was not very effective in seeds, we replaced it in the pX7 vector with the soybean (*Glycine max*) *7S *(which encodes a seed storage protein) gene promoter which displays seed-specific expression [16]. The new vector with the soybean *7S *promoter was named pX8-FAD2-1i (Figure 1A). We generated 30 X8-FAD2-1i lines which were confirmed to be pure marker-free or partial marker-free (partial genotyping results shown in Additional file 3). Quantitative RT-PCR analysis showed that line X8#34 and X8#291 contained only 0.7% and 1.1% *JcFAD2-1 *transcript in the endosperm compared to that of 35S: GFP control lines (Figure 3A). Since soybean *7S *promoter also has high activity in seed cotyledon of soybean, we checked the *JcFAD2-1 *transcript level in the cotyledons of T1 transgenic *Jatropha*. Quantitative RT-PCR results suggested that the soybean *7S *promoter was also active in *Jatropha *cotyledons as much lower levels of *FAD2-1 *transcript accumulated in T1 cotyledons compared with that of the control (Figure 3B). As expected, there was no significant change of *FAD2-1 *transcript levels in vegetative organs such as leaves (Figure 3C).

GC analysis showed that oleic acid accumulation was as high as 77.4% and 74.7% of the total fatty acid content in the T1 endosperm of lines #34 and #291, respectively (Figure 3D; Additional file 4). The linoleic acid levels reduced to less than 5% of the total fatty acid content in these lines. Moreover, the stearic acid level also slightly reduced from 7.7% to 5.4% to 5.7% of the total fatty acid in these transgenic *Jatropha*. There was no marked difference in C16 fatty acids composition between transgenic lines and control plants. The total unsaturated fatty acids (oleic and linoleic) in control and transgenic *Jatropha *endosperms was estimated to be about 78% to 79% of the total fatty acid. In lines #34 and #291, almost all of the unsaturated fatty acids was stored as oleic acid. There was no obvious difference in the total oil content between line #34 endosperm and control endosperm (Figure 3E). Consistent with no changes on gene expression level in leaf (Figure 3C), there was no significant difference in fatty acid profile of true leaves between X8-FAD2-1i lines and controls (Figure 3F,Additional file 4). These data further confirmed the seed-specific high oleic acid accumulation in transgenic lines.

To further analyze the possible side-effect of the *FAD2-1 *RNAi on the fatty acid biosynthesis pathway, we determined endosperm transcript levels of several key fatty acid genes such as *KASII, FATB *and *SAD*. No significant difference between transgenic lines (#34 & #291) and control was found with respect to the expression of these genes implicated in fatty acid biosynthesis (data not shown). This was in agreement with the similar level of oil content between transgenic and control endosperms (Figure 3E).

**Figure 4:**
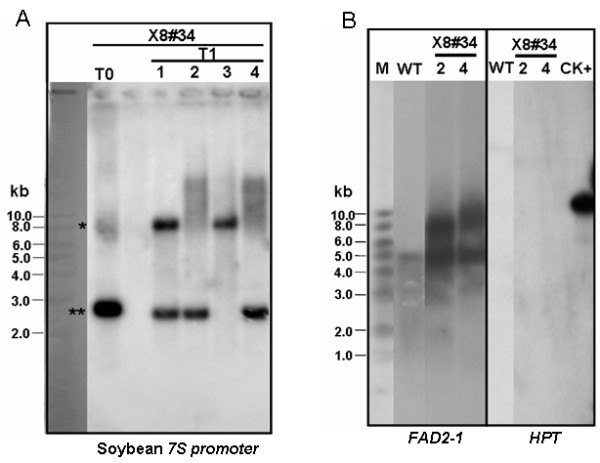
**Southern blot analysis of T0 and T1 plants from X8-FAD2-1i #34 line**. **(A) **Total genomic DNA were digested with *Xho*I and hybridized with soybean *7S *promoter probe. *Shows the positive genomic bands containing marker; **shows the positive genomic bands that are marker free. **(B) **Total genomic DNA were digested with *Eco*RV and *Xba*I and hybridized with *FAD2-1 *probe (left panel). The same membrane was stripped and hybridized with *HPT *probe (right panel). The signals shared between wild-type and the transgenic lines represent the endogenous *JcFAD2-1 *sequence. Each of the transgenic lines also shows one extra band corresponding to the transgene insert.

We performed Southern blot analysis on line #34 to determine the complexity of the transgenic locus. Using PCR-based genotypic analysis, we observed that line #34 was a chimeric marker-free plant (Additional file 3). There is only one *Xho*I site in the pX8-FAD2-1i vector (Figure 1A). In case of a single copy insertion in line #34, we would expect two bands with a size difference of around 5.7 kb due to the partial Cre-lox recombination event. Therefore, the total genomic DNAs of T0 and T1 plants were digested with *Xho*I and probed with soybean *7S *promoter. The Southern blot data (Figure 4A) showed two bands with a size difference of around 5 to 6 kb in #34 T0 plants and these two bands segregated in T1 plants (lines #34-1 to #34-4). T1 plants #34-2 and #34-4 contained a single copy of the transgene and should have been marker free, whereas #34-1 was a chimera and #34-3 still contained the marker. To analyze whether #34-2 and #34-4 were indeed marker free, we further treated the total genomic DNA of these two T1 plants with *Eco*RV and *Xba*I, and hybridized it with FAD2-1 probe. Beside the endogenous 5 kb band from the *JcFAD2-1 *genomic locus according to its genomic DNA sequence, an extra band was found in plants #34-2 and #34-4 (Figure 4B) which was absent in *Jatropha curcas *MD isolate (Jc-MD) wild-type control plant. We stripped the membrane and hybridized it with an HPT probe. No signal was detected in the DNA derived from these two transgenic plants (Figure 4B). These results confirmed that #34-2 and #34-4 plants were indeed marker-free transgenic *Jatropha*.

### Effect of oleic acid manipulation on agronomic traits of the plant

We checked whether there were any negative effects of high oleic acid on agronomic traits under greenhouse conditions. Four T0 trees with high oleic acid trait (oleic acid content > 70% of total fatty acid in T1 seed, including X8#34 and X8#291), 16 T0 transgenic trees with non-high oleic acid and two control wild-type *Jc*-MD plants were monitored for the following agronomic traits: plant height, number of primary branches after one trimming, diameter of main stem, seed number per fruit and seed number per tree after transplanting (September 2009 to June 2011). Previous studies have shown that *Jc*-MD was the earliest to flower and the most productive cultivar in field trials [17]. Under our greenhouse conditions, it took almost one year to flower and there was an average of 12 seeds per tree with an average seed weight of 330 mg, which was comparable to T0 high oleic plants and T0 non-high oleic plants as shown in Table 1. These preliminary data collected from T0 plants under greenhouse conditions indicated that high seed oleic acid does not show negative effects on other *Jatropha *agronomic traits.

**Table 1:** Agronomic traits comparison between wild-type and T0 X8-FAD2-1i transgenic trees

	Height^a ^(cm)	Diameter of main stem^a ^(mm)	Number of primary branches^a^	Seed number per fruit	Seed number per tree	Seed weight per seed (mg)
WT	108.0 ± 13.0 (2)	32.0 ± 2.0 (2)	4.5 ± 0.5 (2)	2.2 ± 0.1 (12)	12.5 ± 0.5 (2)	336.0 ± 27.3 (25)
HO	105.0 ± 5.8 (4)	30.0 ± 1.7 (4)	6.3 ± 1.9 (4)	2.1 ± 0.2 (26)	13.5 ± 3.7 (4)	327.7 ± 20.9 (55)
Non-HO	109.4 ± 7.8 (16)	30.7 ± 1.7 (16)	6.1 ± 0.7 (16)	2.1 ± 0.1 (136)	10.2 ± 3.4 (16)	322.0 ± 23.5 (286)

^a^Measured and recorded in May 2011. The data are presented as average ± standard error (n), where n is the number of fruits, trees or seeds used in the statistical analysis. WT: Wild-type; HO: high-oleic-acid; non-HO: non-high-oleic-acid trait.

## Discussion

Here, we describe the generation of transgenic *Jatropha *plants that accumulate high levels of oleic acid in seed by knockdown of *JcFAD2-1 *expression. Some of the characterized transgenic lines showed drastic changes in the fatty acid composition of seeds. Line X8#34 had 77.4% oleic acid and 2.3% linoleic acid in seeds in contrast to 36.7% oleic acid and 41% linoleic acid in 35S:GFP control seeds. Based on the published results of other crops [2,4], we expect the altered ratio of oleic and linoleic acid will confer desirable properties on the resulting biodiesel. Reports have estimated the CN value of *Jatropha *biodiesel to be within the range of 43.3 to 51 [18,19]. Bamgboye and Hansen [20] developed an equation which establishes the general dependence of CN on FAME composition of biodiesels. Based on this CN equation and the seed fatty acid composition of transgenic and 35S:GFP control plants, we deduced the theoretical CN value of *Jatropha *control biodiesel to be 52.6, which increased to 60.2 in the X8#34 transgenic line. There was also a predictable relative lower NO_x _emission for high oleic acid *Jatropha *biodiesel as suggested by the experimental data on high oleic acid soybean biodiesel [2,4]. Oxidative stability is another key issue for biodiesel. Neat *Jatropha *biodiesel exhibits an oxidation stability of 3.95 hours [21]. As noted before, methyl esters of linoleate are 49 times more reactive than oleate esters [3]; therefore, the oxidation stability of high oleic acid *Jatropha *will be drastically increased. There are no obvious changes in the proportion of saturated fatty acids in the high oleic acid *Jatropha *oil, thus the cold flow properties, cloud and pour points of biodiesel derived from this oil might be the same as the control biodiesel.

The downregulation of *FAD2-1 *using RNAi technology was quite specific as no changes in *FAD2-2 *expression were observed in the leaves, endosperms and cotyledons (Figures 2 and 3). The low sequence similarity (72.9%) between these two transcripts may generate insufficient quantities of secondary siRNAs [22] to trigger *FAD2-2 *transcript degradation as well. The level of oleic acid accumulation was inversely proportional to the *FAD2-1 *transcript level, suggesting that FAD2-1 is the major enzyme responsible for converting oleate to linoleate in both vegetative organs and seeds of *Jatropha*. Since *JcFAD2-2 *played no significant role in normal fatty acid biosynthesis process when it was knocked down by VIGS, a gene duplication event might have occurred for FAD2 to generate another unusual fatty acid enzyme other than oleate desaturase during the early evolution stage of Euphorbiaceae, at least for *Jatropha *(JcFAD2-2), castor bean (RcFAH12), tung tree (VfFADX) and cassava (MeFAD2-2). It will be interesting to test the enzyme activity of JcFAD2-2 and other FAD2-like proteins from other Euphorbiaceae to see whether they possess some novel enzyme activities to produce unusual fatty acids for potential industrial usage.

Reports in soybean show that genotypes obtained from conventional breeding possessing a lesion in one of the *FAD2-1 *genes had mid-oleic acid level traits (30% to70%) but lower yield [23,24]. The main reason that these mid-oleic genotypes have lower yield is most likely due to the weaker environmental adaptation ability of these FAD2-1 mutant plants [7]. The lesion in FAD2-1 alters the fatty acid profile of membranes in vegetative tissues. This may impact membrane fluidity and, in turn, affect the plant's ability to respond to temperature and other environmental changes, ultimately leading to a reduction in yield [7]. Downregulation of *FAD2-1 *in a seed-specific manner in soybean resulted in seeds with greater than 80% oleic acid without compromising its agronomic performance [2]. Similarly in *Jatropha*, seed-specific silencing of *FAD2-1 *did not impose any adverse effect on *Jatropha *vegetative growth, as there was no change in the fatty acid profile in vegetative tissues of T0 (Figure 3F) and T1 (data not shown) plants in greenhouse conditions. Nevertheless, these high oleic acid level transgenic lines need to be further characterized through field trials, which we are planning to conduct. With the proposed field test, we will be able to collect enough seed materials for extensive analysis of diesel properties.

To further improve the biodiesel quality by increasing oleate level in seed, one possible method is to simultaneously downregulate *FAD2-1 *and *FATB*, the gene which encodes palmitoyl acyl-acyl carrier protein (ACP) thioesterase B (FATB). FATB removes the acyl-ACP from 16:0-ACP, thereby terminating fatty acid elongation. This enzyme has been shown to be important for the synthesis of saturated fatty acids, such as 16:0 and 18:0 [25]. *Jatropha FATB *had also previously been functionally identified [8,26]. When *FATB *is downregulated, a larger amount of fatty acid can further elongate to C18 and, consequently, both the level of oleate and the proportion of total unsaturated fatty acid can be increased. Biodiesel derived from such transgenic seeds should have improved cold flow properties and cloud and pour points because of the increased unsaturated fatty acid level.

The genomic sequence of *JcFAD2-1 *identified here can be used in marker-assisted selection to identify genetic markers which can be further associated with oleic acid variation in either intra-species or inter-species *Jatropha *breeding efforts. At least one single nucleotide polymorphisms in the *JcFAD2-1 *coding region has been identified (Chunming Wang and JY, unpublished data). With the availability of the whole genome sequence of *J. curcas *[11] and more molecular markers that could be generated by new-generation sequencing technology, isolation of *Jatropha *with high oleic acid contents in seeds by marker-assisted selection should become feasible.

The marker-free transgenic method used in this study can be used to manipulate several important agronomical traits in *Jatropha*, for example, increasing fruit numbers by the manipulation of hormone biosynthesis [27]. Together with the availability of the whole genome sequence of *Jatropha *and rapid gene functional analysis tools like VIGS, this plant can be transformed into an ideal sustainable energy crop in the tropical and subtropical regions.

## Conclusions

We identified three putative *JcFAD2 *genes through genome-wide analysis. Using hairpin RNAi technology, we generated marker-free transgenic *Jatropha *plants with seed-specific *JcFAD2-1 *suppression. The fatty acid composition of this transgenic *Jatropha *oil shifted from 37% oleic acid and 41% polyunsaturated fatty acids to more than 78% oleic acid and less than 3% polyunsaturated fatty acids. These changes in fatty acid composition should lead to better *Jatropha *biodiesel properties. This is the first successful report on genetic modification of key agronomic traits in *Jatropha *and the use of a marker-free system to develop it. We believe that this study will greatly assist in the biotechnological improvement of various *Jatropha *traits, such as oil yield, stress resistance and oil quality and also for related biofuel crops such as castor bean.

## Methods

### Explant material for transformation

Seeds were obtained from the *J. curcas *(*Jc*-MD) elite plants which were pre-selected by Y Hong and C Yi [17]. The seeds were germinated on^1/2^Murashige and Skoog salt medium. Cotyledons were harvested from five- to seven-day-old seedlings, cut into small pieces (5 × 5 mm) and used as explants.

### *Jatropha *transformation procedure

The detailed transformation protocol has previously been described [28]. In brief, the protocol consisted of four steps; co-cultivation, shoot regeneration, shoot elongation and rooting.

#### Co-cultivation

Small cotyledons pieces were incubated with *Agrobacterium *cells harboring the target expression cassette in 20 mL of medium II for 10 to 20 minutes at 25°C. Explants were then transferred to the co-cultivation medium for two to three days at 22°C in the dark. Then, the co-cultivation explants were rinsed several times with sterile water followed by one wash with 300 mg/L cefotaxime. The cotyledon tissues were blotted dry on a pad of sterilized paper to remove excess surface water. The explants were then placed on a callus formation medium and transferred to darkness at 25 ± 1°C for three weeks. Under this condition, the untransformed explants usually turned brown.

#### Shoot regeneration

Explants with newly emerged hygromycin-resistant callus were transferred into shoot regeneration medium I for three weeks at 25°C under 16-hour light (100 μmol/m^2^/s)/8-hour dark cycles. During this period, any shoots regenerated from callus (about 35% to 40%) would be transferred to shoot regeneration medium II. The transformed hygromycin-resistant regenerated shoots about 2 to 3 mm were transferred to β-estradiol induction medium without hygromycin to induce marker excision. After two weeks of induction, shoots were transferred back to regeneration medium II without hygromycin. Any callus with no regenerated shoots were transferred to shoot regeneration medium III for further regeneration.

#### Shoot elongation

After four weeks, the regenerated shoots were transferred into shoot elongation medium for elongation and bud multiplication

#### Rooting

Elongated shoots of about 2.5 cm were rooted on a rooting medium. Normally, more than one month was required to obtain roots. An alternative method was to use grafting to increase the plant survival rate. Elongated shoots were used as scions for grafting onto non-transgenic rootstocks. Healthy and vigorously growing *Jatropha *plants were chosen to be rootstocks. Both scions and rootstocks were cut into the cambium region so that phloem tissues from both parts connected after joining. The graft joint was then wrapped with parafilm and secured with tape. The grafted *Jatropha *plants were maintained under low light intensity (10 to 20 μmol/m2/s) and 85% humidity for seven days.

### Transgenic plasmid construction and materials

The pCAMBIA1300-derived vector which carried a *35S-GFP *gene cassette was used for *Jatropha *transformation. Transgenic lines (called 35S:GFP lines) were confirmed using gene markers, fluorescence and protein gel analysis.

To generate the β-estradiol chemical-regulated inducible *JcFAD2-1 *RNAi lines, we used a gene-specific 862-bp fragment corresponding to the coding region (nt 85 to 946) of the *JcFAD2-1 *cDNA. This cDNA fragment was PCR-amplified with forward primer 5'-ATCACTCGAGCCACCATTCACACTTGGTCAG-3' and reverse primer 5'-GTATAAGCTTCATGAGTGTCTGTAATGTTATG-3'. The PCR fragment was inserted in the sense orientation into the *Xho*I/*Hind*IIII sites of a pSK-int vector as described previously [15]. Another fragment, amplified with forward primer 5'-CAATAACTAGTACCATGGGTGCCGGTGGCAGAATG-3' and reverse primer 5'-TATTGGATCCGGAAACTTGTTTTTGTACCAGAACAC-3', was subsequently placed in the antisense orientation into the *Bam*HI/*Spe*I sites of pSK-int-sense FAD2-1 to form pSK-int-FAD2-1 RNAi. Finally, the entire RNAi cassette comprising the sense and antisense fragments interspersed by the *actin II *intron was excised from pSK-int using the flanking *Xho*I/*Spe*I sites and inserted into the *Xho*I/*Spe*I site of pX7-GFP vector, yielding the construct pX7-FAD2-1i.

To generate seed-specific *JcFAD2-1 *RNAi lines, we replaced the G10-90 promoter in pX7-GFP with the soybean seed storage protein *7S *gene promoter. The resulting vector was called pX8-GFP. The entire *FAD2-1 *RNAi cassette in pSK-int vector was inserted into pX8-GFP to substitute the GFP coding region and hence the construct pX8*-*FAD2-1i was formed.

Transformants were selected using PCR-based genotypic analysis with either P1 and P4 primers (for X7-FAD2-1 RNAi lines) or P7S and P4 primers (for X8-FAD2-1 RNAi lines), together with primer pairs for the *HPT *gene. Additional file 5 shows the sequence information of these primers. Independent lines (X7#79, X7#170 from X7-FAD2-1i; X8#34, X8#291 from X8-FAD2-1i) were confirmed by the analysis of the expression of *FAD2-1 *and fatty acid composition in endosperms of individual seeds. Plants were grown in a greenhouse under natural photoperiods and ambient temperature (ranged from 25 to 35°C) in Singapore.

### Fatty acid analysis

Total lipid was extracted from 100 mg fresh *Jatropha *leaves as previously described [8]. The outer seed coat was removed from dried *Jatropha *seeds. The seeds were surface sterilized for 1 minute with 75% (v/v) ethanol, immersed in 10% (v/v) H_2_O_2 _for 1 hour, rinsed with sterile water two times, and finally immersed in sterile water overnight at 28°C in darkness for 24 hours. The seed endosperm was carefully separated from the embryo. The dry endosperm part was ground to fine powder and the lipids were extracted with hexane three times. The combined supernatant was transferred to a glass vial and the hexane was evaporated with a flow of dry nitrogen gas at 50°C. The weight of the raw oil was determined and the oil content was recorded as the ratio of raw oil to dried endosperm weight.

About 10 mg of lipid was transmethylated with 3N methanolic-HCl (Sigma, St. Louis, MO, USA) plus 400 μL 2,2, dimethoxypropane (Sigma). The resultant FAMEs were separated by GC and detected using GC Agilent 6890 (Agilent, Santa Clara, CA, USA) employing helium as the carrier gas and DB-23 columns for components separation. The GC analysis was performed at 140°C for 50 seconds and 30°C per minute ramp to 240°C, and the final temperature was maintained for 50 seconds. Peaks were identified based on their retention times compared with a FAME reference mixture (Sigma). The fatty acid composition value included in the analyses was calculated based on the peak area percentage of total fatty acids in three biological replicates. The data were presented as mean**s **± standard deviations.

### RNA extraction and analysis

The 100-mg leaf or endosperm samples were ground to fine powder in liquid nitorigen and extracted with plant RNA purification reagent (Invitrogen, Carlsbad, CA, USA). RNA concentration was measured by Nanodrop (Thermo Scientific, Wilmington, DE, USA). Moloney Murine Leukemia Virus Reverse Transcriptase (Promega, Madison, WI, USA) was used for RT reactions. Real-time PCR was performed with Power SYBR*^® ^*Green PCR Master mix (Applied Biosystems, Foster City, CA, USA) and run in ABI7900HT. All samples were run in triplicate and the data were analyzed with RQ manager at a pre-set threshold cycle value (Applied Biosystems). The *Jatropha rbcL *transcript served as an internal control for leaf RNA samples while the *Jatropha α-tubulin *transcript served as an internal control for seed RNA samples. Threshold cycle values included in the analyses were based on three biological replicates, with three technical replicates for each biological sample. Standard deviation was calculated based on the three biological replicates. See Additional file 5 for PCR primer sequences.

### Southern blot analysis

Total genomic DNA was isolated from the leaves of glasshouse-grown transgenic or control plants by the cetyltrimethylammonium bromide method [29]. Genomic DNA was digested with restriction enzymes and separated on 0.8% agarose gels. The gels were processed and transferred to a nylon Hybond-N^+ ^membrane (GE Biosciences, Buckinghamshire, UK) following standard procedures [30]. Membranes were hybridized with *7S *promoter, *HPT *or *FAD2-1 *open reading frame probes. The probes were labeled with [α-32P]-deoxycytidine triphosphate by random prime synthesis using Amersham Rediprime II Random Primer Labelling System (GE Biosciences). Hybridization was performed overnight at 42°C using the ULTRAHyb-Oligo hybridization buffer (Ambion, Austin, TX, USA) and signals were detected by autoradiography.

### Plant growth condition, agronomic traits collection and statistical analysis

All the transgenic or control plants were grown in a biosafety level 2 greenhouse according to standard practices. The transgenic and control plants were transplanted into pots (diameter 30 cm) and randomly placed in a greenhouse with a space of 1 m × 1 m for each tree in Temasek Life Sciences Laboratory, Singapore. Plant management, such as fertilization, pesticides spraying, watering and artificial fertilization, was carried out according to normal practices. Half a year after transplanting, the plants were pruned at the height of 50 cm from the ground. The tree height, diameter of main stem and number of primary branches were measured and recorded.

Every fruit and seed of these plants was collected and counted and their weight was taken over the whole experiment. Dry T1 seeds were weighed and endosperms were further analyzed for oil content and oil profile. Four T0 plants with > 70% oleic acid content were grouped as high-oleic-acid plants and the other 16 T0 plants with normal oleic acid content (37% to 42%) were grouped as non-high-oleic-acid plants. The data were analyzed with Student's *t*-test and presented in Table 1 as means ± standard errors.

## Abbreviations

ACP: acyl carrier protein; bp: base pairs; CN: cetane number; Jc-MD: Jatropha curcas MD isolate:; FAD: fatty aced desaturase; FAH12: Fatty acid hydroxylase 12; FAME: fatty acid methyl ester; FATB: palmitoyl acyl-acyl carrier protein thioesterase B; GC: gas chromatography; GFP: green fluorescent protein; JcFAD2: *Jatropha curcas *microsomal delta 12 fatty acid desaturase; JcFATB: *Jatropha curcas *palmitoyl acyl-ACP thioesterase B; kb: kilobases; NO_x_: nitrogen oxides; PCR: polymerase chain reaction; RNAi: RNA interference; RT: reverse transcriptase; siRNA: small interfering RNA; VIGS: virus-induced gene silencing.

## Competing interests

Two patents about the gene and the methods used in this report have been filed by Temasek Life Sciences Laboratory.

## Authors' contributions

JY designed the experiments, analyzed the data and drafted the manuscript. HZM and YNB did the *Jatropha *transformation. JQ, CW, SQG, YWS and YFG performed the vector construction, genotyping, molecular analysis and fatty acid analysis experiments. All authors read and approved the final manuscript.

## Supplementary Material

Additional file 1 Sequence information of *JcFAD2-1 *and *JcFAD2-2* Click here for file

Additional file 2 **Comparison of His box 3 from plant FAD2-like enzymes and expression profile of *JcFAD2-1 *and *JcFAD2-2*. (A**) Comparison of His box 3 from plant FAD2-like enzymes. The amino acid of enzyme active center His box 3 is underlined. *Arabidopsis thaliana *FAD2 (AtFAD2) [GenBank:NP_187819]; tung tree (*Vernicia fordii*) FAD2 (VfFAD2) [GenBank:AAN87573]; castor bean (*Ricinus communis*) FAD2 (RcFAD2) [GenBank:XP_002530704.1] and castor bean hydroxylase (RcFAH12) [GenBank:U22378]; tung tree bifunctional conjugase/desaturase (VfFADX) [GenBank: AAN87574]; cassava (*Manihot esculenta*) FAD2-1 (MeFAD2-1) [GenBank: FF536755] and FAD2-2 (MeFAD2-2) [GenBank: DB954105]; *Jatropha curcas *JcFAD2-1 [GenBank: JN544421] and JcFAD2-2 [GenBank: JN544422]. **(B) **Expression profiles of *JcFAD2-1 *and *JcFAD2-2 *genes in *Jatropha *leaf, root and several endosperm development stages. Three weeks, five weeks, seven weeks and eight weeks after fertilization (3WAF, 5 WAF, 7 WAF and 8 WAF) corresponding to the early, middle, late and mature stages of *Jatropha *fatty acid biosynthesis. The expression of *J. curcas ubiquitin-like *(*JcRUBL*) served as control for equal loading. *JcFAD6, Jatropha curcas fatty acid desaturase-6*. Click here for file

Additional file 3 **Genotyping of X7-JcFAD2-1i (A) and X8-JcFAD2-1i (B) plants. (A**) Upper DNA gel shows the genotyping result of line 1-26 with *HPT *gene primer pair for X7-JcFAD2-1i. Lower DNA gel shows the result of line 1-26 with marker-free primer pair (P1+P4). **(B) **Upper DNA gel shows the genotyping result of line 25-49 with *HPT *gene primer pair for X8-JcFAD2-1i. Lower DNA gel shows the result of line 25-49 with marker free primer pair (P7S+P4). Note: *indicates one example of a transgenic chimeric line containing cells that carried the marker as well as cells that were marker free; **indicates one example of a line which was completely marker free. Click here for file

Additional file 4 **Gas chromatography analysis of fatty acid methyl esters isolated from endosperm (A) and leaf (B) of 35S:GFP (CK-) plant (upper panel) and X8#34 T1 plants (lower panel)**. Click here for file

Additional file 5 **Primers used in this research**. Click here for file
