# Supplementary material for: Development of marker-free transgenic Jatropha plants with increased levels of seed oleic acid
Source: Biotechnol Biofuels. 2012 Feb 29;5:10. doi: 10.1186/1754-6834-5-10 (PMC3316142; doi:10.1186/1754-6834-5-10)
Supplement: Additional file 1 — Sequence information of JcFAD2-1 and JcFAD2-2 [file 1754-6834-5-10-S1.DOC]

>JcFAD2-1

atgggtgccggtggcagaatgtctgttcctccttcccccaagaagttggaagctgaggtcttgaaacgagttccatactcgaagccaccattcacacttggtcaggtcaagaaagctatcccacctcattgtttccagcgttctgttctccgctcattctcgtatgttgtttatgacctgacccttgcctttatcttttattatgttgccaccaattacttccacctccttcctcaacccctctcttatgtggcctggccaatttactggtccctccaaggctgtgtcctcactggcatttgggttatagcacatgagtgtgggcatcatgcctttagtgactatcaatggcttgatgacatagttggccttctcctccattcctgtctccttgtcccttacttttcatggaaacatagccaccgccgtcatcactctaacaccggttcccttgagcgagatgaagtatttgtccctaaaaagaaatccaacatccgctggttctccaaataccttaacaacctaccaggccgcctattcactcttaccataacacttgcccttggctggccgctatacctagcatttaatgtttcaggcaggcattatgaccgatttgcctgtcactttgacccatatggccctatctacaatgatcgcgagcgaactgagatattcatttctgatgctggtgttcttgctgtcacttatggtctctaccgtcttgctctagcaaagggctttgcttgggttatttgcgtttatggagtacctttgttagtggtgagtgcatttcttgttatgatcacatatctgcaacatactcatccttcattgccgcattatgattcttctgagtgggattggctgagaggcgcgctcgcaactgttgatagagattacggaatcttgaacaaggtattccataacattacagacactcatgtagctcaccatttgttttctacaatgccacattatcatgcaatggaggctacaaatgccataaaaccaattctgggagaatattaccaattcgacaggactcctttcttcaaggcaatgtggagagaggcaaaagagtgcatttacgttgaaccagatgatgctgatcaaagcagaggtgtgttctggtacaaaaacaagttttga

>JcFAD2-1

mgaggrmsvppspkkleaevlkrvpyskppftlgqvkkaipphcfqrsvlrsfsyvvydltlafifyyvatnyfhllpqplsyvawpiywslqgcvltgiwviahecghhafsdyqwlddivglllhscllvpyfswkhshrrhhsntgslerdevfvpkkksnirwfskylnnlpgrlftltitlalgwplylafnvsgrhydrfachfdpygpiyndrerteifisdagvlavtyglyrlalakgfawvicvygvpllvvsaflvmitylqhthpslphydssewdwlrgalatvdrdygilnkvfhnitdthvahhlfstmphyhameatnaikpilgeyyqfdrtpffkamwreakeciyvepddadqsrgvfwyknkf

>JcFAD2-2

atgtttaataatgaatctgcagagaagcttaacagaacaatgggagccggtggccaaaaaactgctgtcctcgtcagcagtaagttcaaggaaatggaaaccaacagacgcctgaagcgagttccacacacaaaacctccattcactcttggccaaatcaaacaagccatcccatcccattgctttaaacgctcccttctccgctctttctcctaccttgtttatgacctctctttaagctctctcttctactacattgccgctagctacttccatcttctcccttctccgatctcctacattgcttggcccatctactggactctccagggctgcactctcactggtgtttgggtcattgctcatgaatgcggccaccatgcttttagtgactatcaatgggttgatgacactgttggcctaattctccactcttcacttcttgttccttatttttcatggaaaattagccatcgtcgccatcactccaacaccggttccattgaacgtgatgaggtctttgtccccaaattcaagtctagaatcccttggtattcccagtacctcaataatccactaggccgagctttagccctcgcagccacactcacggtcggctggccgttgtacttagccttcaatgtctctggacgaccttataatcggtttgcttgtcactttgatccttctggacctatatattctgatagagaaaggcttcagatttacatttctgacattgggattttcgctgcaacttatgtgctctatcagattgccatggcaaaagggttagcttggctgatatctatttatgggataccattgcttattgttaatgcttttcttgtgacaatcacatatttgcagcacactcaccctgcattgccacactatgactcgtccgaatgggattggctccggggagctttgtcgacagtggatagagattatggggtgttgaataaggttttccataatattacagacactcatgtaacccaccatctcttctctacaatgcctcattatcatgcaatggaggccactaaagcaatcaagcctatattgggcgagtattatcagtttgatggcactccgattcttatggcgctctggagggaggccaaggagtgcctgtttgtcgagccagaagagggaggtcccaaccgaggagttctctggtatggaaataagtattaa

>JcFAD2-2

mgaggqktavlvsskfkemetnrrlkrvphtkppftlgqikqaipshcfkrsllrsfsylvydlslsslfyyiaasyfhllpspisyiawpiywtlqgctltgvwviahecghhafsdyqwvddtvglilhssllvpyfswkishrrhhsntgsierdevfvpkfksripwysqylnnplgralalaatltvgwplylafnvsgrpynrfachfdpsgpiysdrerlqiyisdigifaatyvlyqiamakglawlisiygipllivnaflvtitylqhthpalphydssewdwlrgalstvdrdygvlnkvfhnitdthvthhlfstmphyhameatkaikpilgeyyqfdgtpilmalwreakeclfvepeeggpnrgvlwygnky
