# Supplementary figures and images for: Development of marker-free transgenic Jatropha plants with increased levels of seed oleic acid
Source: Biotechnol Biofuels. 2012 Feb 29;5:10. doi: 10.1186/1754-6834-5-10 (PMC3316142; doi:10.1186/1754-6834-5-10)

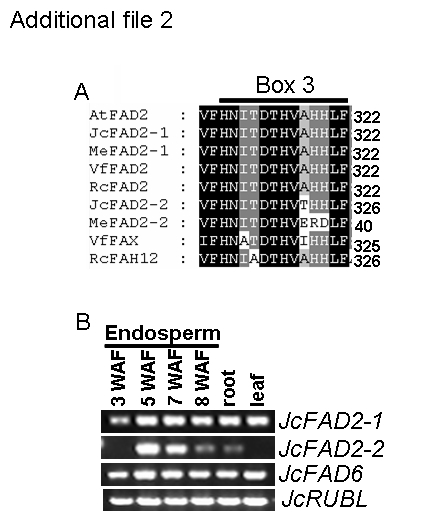

Supplement: Additional file 2 — Comparison of His box 3 from plant FAD2-like enzymes and expression profile of JcFAD2-1 and JcFAD2-2. (A) Comparison of His box 3 from plant FAD2-like enzymes. The amino acid of enzyme active center His box 3 is underlined. Arabidopsis thaliana FAD2 (AtFAD2) [GenBank:NP_187819]; tung tree (Vernicia fordii) FAD2 (VfFAD2) [GenBank:AAN87573]; castor bean (Ricinus communis) FAD2 (RcFAD2) [GenBank:XP_002530704.1] and castor bean hydroxylase (RcFAH12) [GenBank:U22378]; tung tree bifunctional conjugase/desaturase (VfFADX) [GenBank: AAN87574]; cassava (Manihot esculenta) FAD2-1 (MeFAD2-1) [GenBank: FF536755] and FAD2-2 (MeFAD2-2) [GenBank: DB954105]; Jatropha curcas JcFAD2-1 [GenBank: JN544421] and JcFAD2-2 [GenBank: JN544422]. (B) Expression profiles of JcFAD2-1 and JcFAD2-2 genes in Jatropha leaf, root and several endosperm development stages. Three weeks, five weeks, seven weeks and eight weeks after fertilization (3WAF, 5 WAF, 7 WAF and 8 WAF) corresponding to the early, middle, late and mature stages of Jatropha fatty acid biosynthesis. The expression of J. curcas ubiquitin-like (JcRUBL) served as control for equal loading. JcFAD6, Jatropha curcas fatty acid desaturase-6. [file 1754-6834-5-10-S2.TIFF]

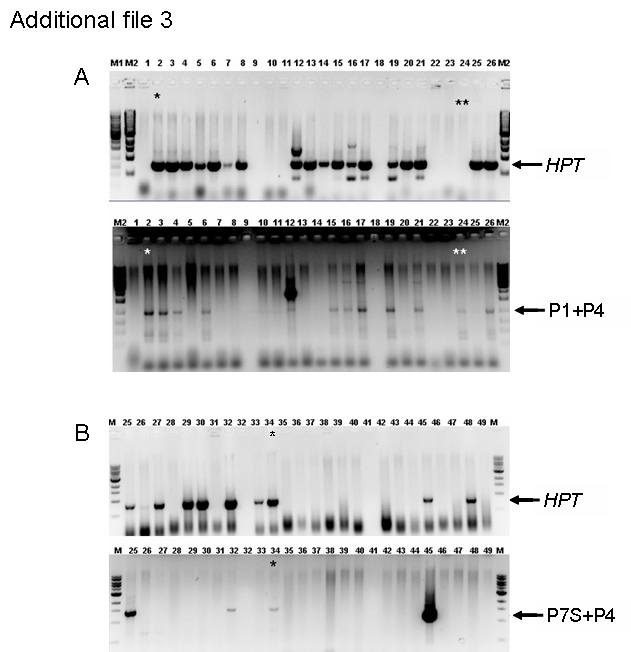

Supplement: Additional file 3 — Genotyping of X7-JcFAD2-1i (A) and X8-JcFAD2-1i (B) plants. (A) Upper DNA gel shows the genotyping result of line 1-26 with HPT gene primer pair for X7-JcFAD2-1i. Lower DNA gel shows the result of line 1-26 with marker-free primer pair (P1+P4). (B) Upper DNA gel shows the genotyping result of line 25-49 with HPT gene primer pair for X8-JcFAD2-1i. Lower DNA gel shows the result of line 25-49 with marker free primer pair (P7S+P4). Note: *indicates one example of a transgenic chimeric line containing cells that carried the marker as well as cells that were marker free; **indicates one example of a line which was completely marker free. [file 1754-6834-5-10-S3.TIFF]

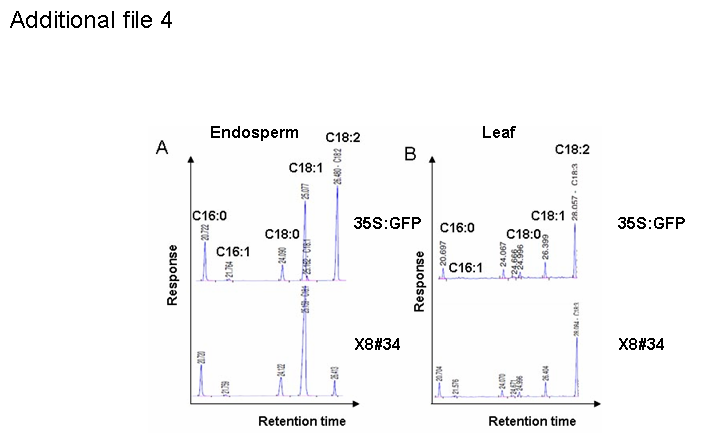

Supplement: Additional file 4 — Gas chromatography analysis of fatty acid methyl esters isolated from endosperm (A) and leaf (B) of 35S:GFP (CK-) plant (upper panel) and X8#34 T1 plants (lower panel). [file 1754-6834-5-10-S4.TIFF]
